# Plasma Hyperosmolality Prolongs QTc Interval and Increases Risk for Atrial Fibrillation in Traumatic Brain Injury Patients

**DOI:** 10.3390/jcm9051293

**Published:** 2020-04-30

**Authors:** Wojciech Dabrowski, Dorota Siwicka-Gieroba, Chiara Robba, Rafael Badenes, Mateusz Bialy, Paulina Iwaniuk, Todd T Schlegel, Andrzej Jaroszynski

**Affiliations:** 1 Department of Anaesthesiology and Intensive Therapy Medical University of Lublin, 20-954 Lublin, Poland; siw@wp.pl (D.S.-G.); mateuszbialy@interia.pl (M.B.); paulina.pawlik89@gmail.com (P.I.); 2 Department of Anaesthesia and Intensive Care, Policlinico San Martino, 16100 Genova, Italy; kiarobba@gmail.com; 3 Department of Anesthesiology and Intensive Care, Hospital Clìnico Universitario de Valencia, University of Valencia, 46010 Valencia, Spain; rafaelbadenes@gmail.com; 4 Department of Molecular Medicine and Surgery, Karolinska Institute, Stockholm, Sweden, and Nicollier-Schlegel SARL, 1270 Trélex, Switzerland; ttschlegel@gmail.com; 5 Department of Nephrology, Institute of Medical Science, Jan Kochanowski University of Kielce, 25-317 Kielce, Poland; jaroszynskiaj@interia.pl

**Keywords:** traumatic brain injury, cardiac arrhythmias, plasma osmolality, osmolar gap, electrocardiography

## Abstract

Introduction: Hyperosmotic therapy with mannitol is frequently used for treatment cerebral edema, and 320 mOsm/kg H_2_O has been recommended as a high limit for therapeutic plasma osmolality. However, plasma hyperosmolality may impair cardiac function, increasing the risk of cardiac events. The aim of this study was to analyze the relation between changes in plasma osmolality and electrocardiographic variables and cardiac arrhythmia in patients treated for isolated traumatic brain injury (iTBI). Methods: Adult iTBI patients requiring mannitol infusion following cerebral edema, and with a Glasgow Coma Score below 8, were included. Plasma osmolality was measured with Osmometr 800 CLG. Spatial QRS-T angle (spQRS-T), corrected QT interval (QTc) and STJ segment were calculated from digital resting 12-lead ECGs and analyzed in relation to four levels of plasma osmolality: (A) <280 mOsm/kg H_2_O; (B) 280–295 mOsm/kg H_2_O; (C) 295–310 mOsm/kg H_2_O; and (D) >310 mOsm/kg H_2_O. All parameters were measured during five consecutive days of treatment. Results: 94 patients aged 18-64 were studied. Increased plasma osmolality correlated with prolonged QTc (*p* < 0.001), intensified disorders in STJ and increased the risk for cardiac arrhythmia. Moreover, plasma osmolality >313 mOms/kg H_2_O significantly increased the risk of QTc prolongation >500 ms. Conclusion: In patients treated for iTBI, excessively increased plasma osmolality contributes to electrocardiographic disorders including prolonged QTc, while also correlating with increased risk for cardiac arrhythmias.

## 1. Introduction 

Hyperosmotic therapy is a standard treatment for cerebral edema (CE) and increased intracranial pressure in patients with traumatic brain injury (TBI) [1,2]. The most common osmotic therapies include mannitol or/and hypertonic saline (HT), which increase the osmotic gradient across the blood–brain barrier (BBB) and enable the shift of water from the brain to the vascular space [2]. The final goal of hyperosmotic therapy is the achievement of plasma osmolality of 300–320 mOsm/kg [1,2]. Plasma osmolality mainly depends on the concentration of solutes dissolved in a solvent, often measured by a cryoscopic reference method [3].

In the clinical practice, electrolytes, glucose, and urea are the most important solutes affecting osmotic activity, and their concentration in one liter of solution is defined as plasma osmolarity. Several popular formulas are used for the calculation of plasma osmolality [4,5]. However, plasma osmotic activity also depends on other substances, such as alcohol, ethylene glycol, and radiology contrast [6,7]. It has been documented that plasma ethanol concentration is linearly correlated with increase in plasma osmolality and that each milligram of ethanol per deciliter contributes to 0.22 mOsm/kg (1‰ = 22 mOsm/kg) [4,5,6,7,8].

The difference between measured plasma osmolality and calculated plasma osmolarity is called the osmolal gap. Normally osmolal gap ranges between −10 to +10, and values higher than 10 mOsm/L suggest the presence of water-soluble osmotically active substances in the blood, whereas values higher than 20 mOsm/L suggest a state of intoxication [7,9,10].

An increase in plasma osmolality as well as osmolal gap may lead to organ damage. For example, hyperosmolality can cause acute kidney injury (AKI) [11,12], and also impair cardiac function, increasing mortality in patients with heart failure [13,14,15]. In fact, hyperosmotic stress promotes cardiomyocyte injury [16], and an increase in plasma osmolality following hyperosmolar contrast administration can cause significant disorders in cardiac repolarisation reflected in electrocardiographic measurement [17,18].

Various electrocardiographic parameters including prolonged QT corrected (QTc) interval and widened spatial QRS-T angle (spQRS-T) are well recognized predictors of life-threatening cardiac arrhythmias and sudden cardiac death [19,20,21,22]. Prolonged QTc interval usually reflects dysfunction of cardiac ion channels with abnormal myocardial repolarization and prolongation of the action potential. Increased spQRS-T angle can in turn quantify the often-excessive difference in the spatial direction between ventricular depolarization and repolarization in those more prone to cardiac arrhythmias. Finally, abnormalities in STJ segment levels often reflect changes in ventricular repolarization driven either by changes in cardiac autonomic regulation and/or resulting from cardiac ischemia.

Previously, we documented a significant prolongation of QTc interval in the first 24 h after TBI [23]. Several authors have suggested that TBI-related cardiac dysfunction is common and the consequence of the dysregulation of the central autonomic system [24,25]. However, an increase in plasma osmolality alone can also lead to cardiac dysfunction and increase mortality rate [14,16]. Therefore, we hypothesized that increase in plasma osmolality alone, following hyperosmotic treatment with mannitol, might also be an independent risk factor for cardiac arrhythmias in patients with TBI. The aim of this study was to explore potential associations between changes in plasma osmolality with changes in QTc interval, spQRS-T angle, and cardiac arrhythmias during intensive care unit (ICU) stays in patients with isolated TBI.

## 2. Methods

This prospective, observational study was conducted in accordance with the Declaration of Helsinki, with applicable regulatory requirements approved by the Institutional Review Board and the Bioethics Committee of Medical University at Lublin, Poland (KE-0254/26/2019). Informed consent was obtained from patients’ legal representatives, as all enrolled patients were unconscious or/and sedated at the moment of the inclusion in the study.

### 2.1. Patient Selection

Adult patients who were treated for severe or moderate isolated TBI (classified according to the Glasgow Coma Score (GCS)), and who required hyperosmotic therapy due to intracranial hypertension, were included in the study. Exclusion criteria were pregnancy, age below 18 years, and patients with active cardiac disease, or cardiac/cardiosurgical history. Moreover, patients with a pre-injury history of endocrine, metabolic, pulmonary, or hepatorenal diseases, as well as drug-intoxicated patients and prior transplant recipients, were excluded.

For the entire duration of the ICU stay, relevant demographic, clinical and laboratory data along with daily assessment of fluid balance, sepsis-related organ failure assessment (SOFA) score, and advanced hemodynamic monitoring variables (obtained with EV1000) were registered in an electronic database, as well as data on mortality at day 28.

Severity of illness on ICU admission was described by the acute physiology and chronic health evaluation (APACHE-II) and trauma scores.

### 2.2. Patient Monitoring and Management

Detailed monitoring and treatment techniques have been previously described [23]. In brief, arterial blood pressures and heart rate were continuously measured. Additionally, hemodynamic variables such as cardiac output/index (CO/CI), stroke volume variation (SVV), systemic vascular resistance index (SVRI), and central venous pressure (CVP) were monitored using EV 1000 platform. Masimo Root monitor (USA) with SEDLine was used for continuous measurement of regional cerebral oxygen saturation (SrO_2_), fronto-temporal electroencephalography, peripheral saturation (SpO_2_) with haemoglobin level and Pleth Variability Index (PVI). Transcranial Doppler (TDC) was performed to measure the velocity of cerebral blood flow. Hyperosmotic therapy included the administration of boluses of 15% mannitol. This treatment was discontinued in patients with osmolality higher than 320 mOsm/kg H_2_O. Blood potassium concentration was measured 5 times per day and eventually corrected using continuous infusion of potassium and magnesium mixture. Fluid administration and vasopressors (norepinephrine) were titrated to obtain SrO_2_ higher than 50% and mean arterial pressure (MAP) higher than 80 mmHg.

Surface 12-lead resting ECGs with derived vectorcardiograms (VCGs) were recorded using a Cardiax device (IMED Co Ltd., Budapest, Hungary). The recordings at each time period were automatically averaged to a single beat, and transformed into three orthogonal leads X, Y, and Z according to the inverse Dower method [26,27]. The projections of the maximum vectors of QRS and T-waves in the frontal, transverse, and left sagittal planes and on the *x*, *y*, and *z* axes were then obtained. Next, the value for the spQRS-T angle, automatically calculated from the maximum spatial QRS and T vectors, as well as the values of the QTc interval, STJ, QRS, and T axes (QRS_ax_, T_ax_) and T amplitudes (T_a_), were obtained directly from the Cardiax commercial software.

### 2.3. Plasma Osmolality, Osmolar Gap, and Time Points

Plasma osmolality was estimated using cryometric measurement. The sample was supercooled to a temperature just below freezing point but estimated in the liquid sample while still in crystallization. A two-phase system was created as an ice crystal solution. Heat of crystallization increases system temperature, which reached the maximum value depending on plasma osmolality, calculated by Osmometr 800 CLG (Trident-Med s.c. Warsaw, Pl). The osmolarity was calculated as: 2 × Na (mEq/L) + glucose (mmol/L) + BUN (mmol/L). Osmolar gap was calculated as the difference between measured plasma osmolality and calculated plasma osmolarity.

All observations were performed at five time points: (1) immediately after the admission to ICU; (2) 24 h after the admission; (3) 48 h after the admission; (4) 72 h after the admission; and (5) 96 h after the admission. The values of QTc, spQRS-T angle and occurrence of tachyarrhythmias were compared with plasma osmolality at each time point. Specifically, the continuous electrocardiographic variables were analyzed in accordance with the following cut-offs for plasma osmolality: (A) <280 mOsm/kg H_2_O; (B) between 280–295 mOsm/kg H_2_O (physiological value); (C) between 295–310 mOsm/kg H_2_O; and (D) >310 mOsm/kg H_2_O. Patients with positive blood alcohol concentration were allocated into fifth group (Alc.) and their electrocardiographic variables were analyzed separately. Data were also analyzed in relation to occurrence of cardiac arrhythmia.

### 2.4. Statistical Analysis

Means and standard deviations (SD) were calculated. Categorical variables were compared using the χ^2^ and Fisher exact test, or χ^2^ with Yates correction when applicable. Student’s unpaired *t*-test was used for variables with normal distribution. For variables with non-normal distribution, the Wilcoxon signed-rank, Mann-Whitney-U, Kruskal–Wallis ANOVA, and post-hoc Dunnett’s multiple comparison tests were used. Additionally, Spearman’s rank correlation tests were used for inter-point and overall comparisons. Linear regression analysis was performed by using the Pearson’s test for variables with normal distribution and the Spearman’s test for variables with non-normal distribution. Multiple stepwise regression analysis was performed to estimate the potential influence of various factors on the changes in spQRS-T angle and STJ. The following independence parameters were entered into the model: fluid balance, osmolar gap, plasma Na^+^ concentration, and dose of norepinephrine. Cut-off points were calculated with the use of receiver-operator characteristics (ROC) with auto-calculated maximum specificity and sensitivity. The power of the statistical tests was assessed by the G*Power test. A *p* < 0.05 was considered significant.

## 3. Results

One hundred and twenty-two isolated TBI patients admitted to the ICU between January and December 2019 were considered for inclusion in the study. Twenty-six patients were excluded due to previous cardiac history (information received from the legal representatives or medical documentation) and two patients were excluded due to the absence of complete data for the analysis. Finally, 94 patients aged 18-64 were included in the study (Table 1).

**Figure 1:**
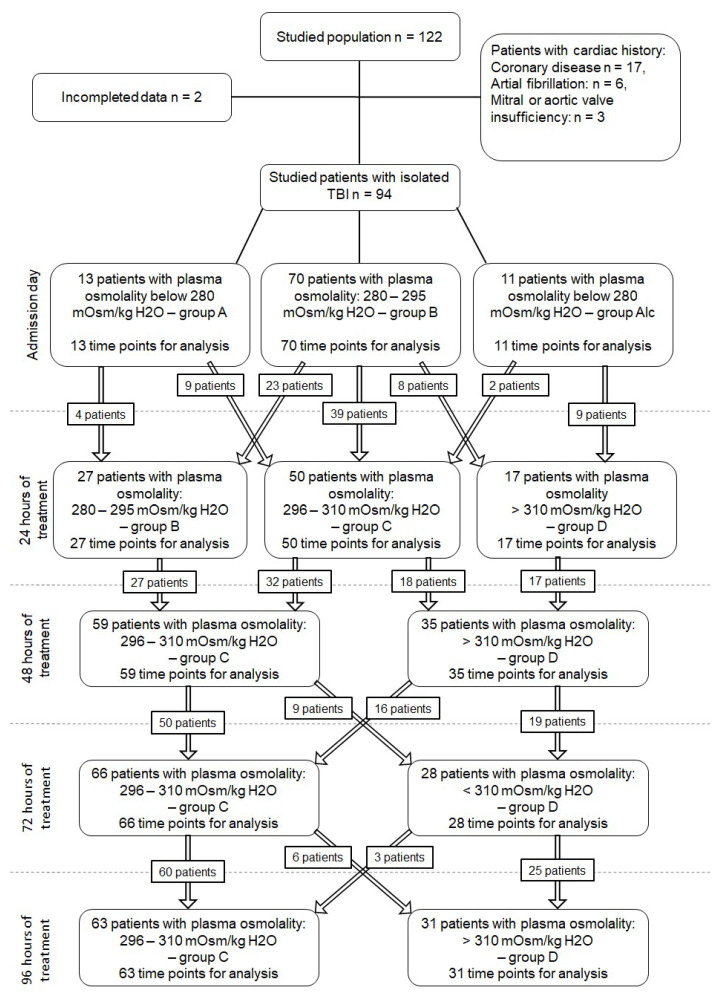
Patient distribution according to plasma osmolality. Taken together, plasma osmolality <280 mOsm/kg/H_2_O was noted in 13 patients on the admission day (13 time points); between 280–295 mOsm/kg H_2_O in 97 patients at least once during the five studied time points (97 time points); between 296–310 mOsm/kg H_2_O in 238 patients at least once during the five studied time points (238 time points); and >310 mOsm/kg H_2_O in 111 patients at least once during the five studied time points (111 time points). Positive blood alcohol was found in 13 patients on admission day.

Correlations between QTc, spQRS-T, STJ, and plasma osmolality were measured at 470 time points (Figure 1). Plasma osmolality below 280 mOsm/kg H_2_O was found in 13 patients, always noted just after the admission to ICU but before the beginning of osmotherapy (group A). Plasma osmolality between 280 and 295 mOsm/kg H_2_O was found in 70 patients without blood alcohol content just after the admission to ICU, again before the beginning of osmotherapy, with such an osmolality range again being noted in 27 patients 24 h after the beginning of mannitol infusion. Plasma osmolality between 280 and 295 mOsm/kg H_2_O was noted at 97 time points (group B). Hyperosmotic therapy with mannitol then increased plasma osmolality. Values ranging between 296 and 310 mOsm/kg H_2_O were then noted at 238 time points, and values >310 mOsm/kg H_2_O at 111 time points (excluding patients with blood alcohol content).

The presence of alcohol in the blood was found in 11 patients just after the admission into ICU, their mean value of plasma osmolality being 322.82 ± 12.67 mOsm/kg H_2_O. Those patients did not receive hyperosmotic therapy until plasma osmolality decreased below 310 mOsm/kg H_2_O.

### 3.1. Plasma Osmolality, Blood Sodium Concentrations, and Osmolar Gap

Plasma osmolality was similar between patients with alcohol intake and those in group D (319 ± 6.13 vs. 322.82 ± 12.67 mOsm/kg H_2_O, *p* = 0.62). The mean blood sodium concentrations were 130.92 ± 5.21, 137.09 ± 2.29, 142.84 ± 3.54, 146.5 ± 3.87 and 142.91 ± 2.39 in groups A, B, C, D, and Alc, respectively. Blood sodium concentration was significantly lower in group A than in groups B, C, D, and Alc (*p* < 0.001). Additionally, blood sodium concentration was lower in group B than in groups C, D, and Alc (*p* < 0.001), and significantly lower in group C than group D (*p* < 0.001). Blood sodium concentration was higher in group D than Alc (*p* < 0.01).

Mean value of the osmolal gap was: 0.9 ± 2.62 [minimum −1.44, maximum 5.22] in group A, 1.26 ± 1.75 [−0.53, 8.17] in group B, 4.41 ± 4.7 [−0.3, 23.2] in group C, 12.1 ± 8.62 [−0.2, 47.8] in group D, and −0.01 ± 6.29 [−0.2, 21.1] in group Alc. An osmolar gap higher than 20 mOsm/L was noted in 2 patients in group C, 20 patients in group D and 1 patient in group Alc. An osmolar gap between 10 and 20 mOsm/L was noted in 34 patients in group C, 42 patients in group D and 2 patients in group Alc. While SpQRS-T angle was significantly wider in patients with osmolar gap higher than 20 mOsm/L than in patients with osmolar gap below 10 mOsm/L (81.6° ± 57.4° vs. 54.3° ± 48.8°, *p* < 0.05), there were otherwise no significant correlations between osmolar gaps and spQRS-T angle or QTc interval in all studied groups.

### 3.2. Plasma Osmolality, Osmolar Gap, and Electrocardiographic Disorders

**Figure 2:**
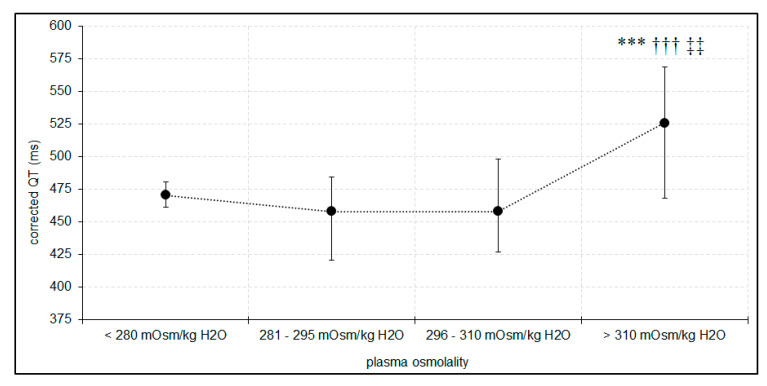
Differences in corrected QT interval (QTc) in relation to plasma osmolality. *** *p* < 0.001—significant difference compared with group C (plasma osmolality 296–310 mOsm/kg H2O), ††† *p* < 0.001—significant difference compared with group B (plasma osmolality 280–296 mOsm/kg H2O), ‡‡ *p* < 0.01—significant difference compared with group A (plasma osmolality below 280 mOsm/kg H2O).

In our cohort, plasma osmolality >310 mOsm/kg H_2_O was associated with prolonged QTc interval, with the highest QTc interval values noted in group D (Figure 2).

**Figure 3:**
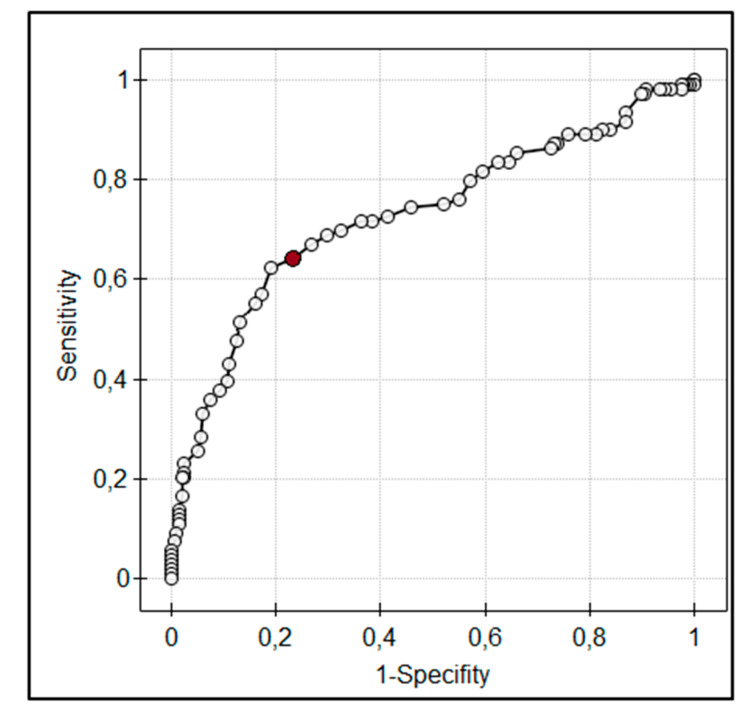
The receiver-operator characteristics (ROC) curve for corrected QT interval (QTc) and plasma osmolality in the study population. Increase in plasma osmolality above 313 mOsm/kg H_2_O increases the risk for QTc prolongation >500 ms.

The value of QTc interval was comparable in group D and Alc (522 ± 64.16 vs. 533.09 ± 49.51, *p* = 0.95). The results of multiple regression analysis showed that QTc was independently associated with plasma osmolality as well as dose of norepinephrine (Table 2). These relationships were not observed in spQRS-T angle analysis. SpQRS-T angles were comparable in all groups and their mean values were 38.33° ± 33.51° in group A, 57.79° ± 51.03° in group B, 53.77° ± 41.42° in group C, 63.7° ± 57.54° in group D, and 58.45° ± 30.11° in group Alc. ROC analysis showed that increased plasma osmolality is a significant risk factor for prolonged QTc with >313 mOsm/kg H_2_O being a cut-off for notable (>500ms) prolongation of QTc (Figure 3). SpQRS-T angle was significantly wider in patients with osmolar gap higher than 20 mOsm/L than in patients with an osmolar gap below 10 mOsm/L (81.6° ± 57.4° vs. 54.3° ± 48.8°, *p* < 0.05).

**Figure 4:**
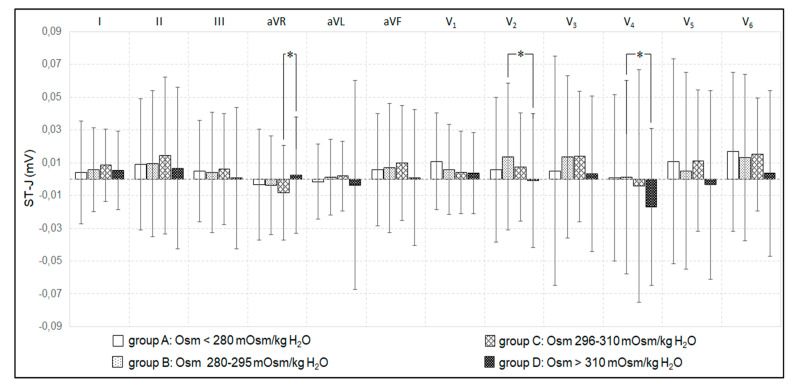
Changes in STJ segments in 12 leads in relation to plasma osmolality. * *p* < 0.05 –significant differences between studied groups.

Increase in plasma osmolality above 310 mOsm/kg H_2_O elevated the STJ segment in lead aVR and depressed it in leads V_2_ and V_4_ (Figure 4). Increase in plasma osmolality inversely correlated with STJ segment in V_6_ lead in groups C (*p* < 0.05, r = −0.22) and D (*p* < 0.05, r = −0.29). In Alc group, STJ segment inversely correlated with plasma osmolality in leads: I (*p* < 0.01, r = −0.78) and V_6_ (*p* < 0.05, r = −0.65), and positively in lead III (*p* < 0.05, r = 0.7).

Atrial fibrillation was noted at 1 time point in groups A and Alc, at 11 time points in group B (mean plasma osmolality was 286.64 ± 4.18 mOsm/kg H_2_O), 16 time points in group C (mean plasma osmolality was 303.5 ± 4.95 mOsm/kg H_2_O) and 26 time points in group D (mean plasma osmolality was 319.98 ± 5.73 mOsm/kg H_2_O). The risk for atrial fibrillation was higher in group D than C (χ^2^ = 8.65, *p* = 0.01, χ^2^ with Yates correction = 7.64, *p* < 0.01) and in group D than B (χ^2^ = 6.54, *p* = 0.05, χ^2^ with Yates correction = 5.61, *p* < 0.05). Additionally, the risk for atrial fibrillation was higher in group C than B (χ^2^ = 5.68, *p* = 0.05, χ^2^ with Yates correction = 4.82, *p* < 0.05). All patients with atrial fibrillation in group D had plasma osmolality higher than 313 mOsm/kg H_2_O. The incidence of atrial fibrillation did not depend on the osmolar gap.

## 4. Discussion

To our knowledge, this is the first study analyzing the effects of hyperosmotic treatment in TBI patients on electrocardiographic changes and cardiac arrhythmias. We documented that the risk of QTc prolongation higher than 500 ms significantly increases when plasma osmolality exceeds 313 mOms/kg H_2_O. Additionally, an increased plasma osmolality by osmotic infusion to above 310 mOms/kg H_2_O can intensify disorders in STJ segment and increase the risk of atrial fibrillation.

Current guidelines recommend hyperosmotic therapy to increase plasma osmolality in patients with cerebral edema, but without exceeding 320 mOsm/kg H_2_O [1,2]. Mannitol infusion at the dose 0.2–1 g/kg body weight is popularly used for hyperosmotic therapy [1,2,28]. Relatively rapid increase in plasma osmolality following mannitol infusion causes intravascular volume expansion, which may lead to heart failure, particularly in patients with cardiac diseases [28]. Mannitol also increases the risk for electrolyte disturbance including hypokalemia and hypomagnesaemia [28,29], which can further enhance the risk for different types of cardiac arrhythmias. In the present study, all patients received continuous infusion with potassium and magnesium, and the plasma potassium concentration was maintained between 4.5–5 mmol/L. The hemodynamic variables were continuously controlled, and intravascular volume expansion or depletion was immediately corrected with diuretics or crystalloids infusion when clinically indicated. Therefore, we can speculate that ECG disorders and cardiac arrhythmias were associated primarily with the increase in plasma osmolality or mannitol than with electrolyte or intravascular volume disorders. Additionally, all our patients had no cardiac history and they did not receive any cardiac medications.

Disorders in plasma osmolality may induce/intensify cardiac failure per se. Experimental models have documented that hyperosmotic stress can promote endoplasmic reticulum stress via increases in the cardiac myocyte calcium content, also a trigger for myocyte apoptosis and hyperglycemia-induced myocyte death [15]. Additionally, an increase in extracellular osmolarity impairs cardiac contraction following disorders in the intracellular calcium homeostasis [30]. All these factors may depress cardiac function in TBI patients.

Importantly, raised plasma osmolality has shown to be an important risk factor for poor outcome in patients with heart failure [13,15,16,31]. Some authors have documented that the increase in plasma osmolality above 310 mOsm kg/H_2_O significantly increases the necessity for use of vasoactive drugs and the risk of death [16,31]. High plasma osmolality is also a strong predictor of both in-hospital and long-term mortality in patients with acute coronary syndrome [32]. In the present study, we documented a significantly prolonged QTc interval in patients with plasma osmolality higher than 310 mOsm kg/H_2_O, and we calculated 313 mOsm kg/H_2_O as a cut-off point for QTc prolongation higher than 500 ms. The incidence of atrial fibrillation was also higher in such patients.

Prolonged QTc interval is a well-established risk factor for life-threatening cardiac arrhythmias [19,20,21,22,32]. QTc value higher than 500 ms increases the probability of cardiac events [32]. Several factors affect QTc interval, and a significant prolongation in QTc interval with sensitization of the myocardium to cardiac arrhythmias including life-threatening ventricular arrhythmias has also been noted in alcohol intoxicated patients [33]. Importantly, these effects strongly depend on the dose of alcohol [34], as alcohol increases plasma osmolality in a dose-dependent manner [6,7,8]. Unfortunately, the effect of plasma hyperosmolality on QTc and cardiac arrhythmias has been poorly recognized. Some authors suggested that hyperosmolality-induced myocardial dehydration was associated with electrocardiography abnormalities and might be one of the mechanisms for the development of Takotsubo cardiomyopathy, a serious cardiac complication in TBI patients [35,36]. Indeed, rapid infusion of hypertonic solution into coronary arteries can significantly prolong QTc and QRS-T-time with subsequent ventricular fibrillation [18]. In the present study, the increase in plasma osmolality prolonged QTc, and the longest QTc was noted in both groups Alc and D. Additionally, cardiac arrhythmias were noted in 26 patients with plasma osmolality higher than 310 mOsm/kg H_2_O and in 1 patient with blood alcohol content. Based on our findings, we can speculate that an increase in plasma osmolality may induce electrocardiographic disorders and sensitize myocardium to arrhythmias; however, this relationship should be confirmed in the further larger studies.

### Limitations

Despite its novel findings, the present study also has several limitations. First, we calculated the plasma osmolarity using one of the simplest formulas (2 × Na + glucose + BUN). However, several formulas have been proposed to calculate plasma osmolarity and osmolar gap may vary depending on the formula used [4,5,8]. Additionally, we did not analyze the relationships between plasma hyperosmolality and the final late outcome measured by Glasgow Outcome Score. Importantly, hypernatremia was the main cause of hyperosmolality in our patients. Han and colleagues have documented that elevated blood sodium concentration following hyperosmotic treatment with hypertonic saline solutions (7.5%) may induce cardiac arrhythmia in hypovolemic patients [37]. However, they noted that all these patients treated with this hypertonic saline solution had low blood potassium concentration, which could induce cardiac arrhythmias. In the present study, all patients received continuous potassium and magnesium infusion, and hypernatremia was noted in 18 patients (72%) with atrial fibrillation in group D and in 7 patients (44%) in group C. In all these patients blood potassium concentration was higher than 4 mmol/L (data not shown). Therefore, we can speculate that hypernatremia may be one of many factors inducing cardiac arrhythmias; however, this relation should be further confirmed.

Another limitation was our inability to relate changes in QTc interval to other medications administered to a minority of patients at baseline that might have also affected QTc interval. It is well recognized that several medications affect QTc interval per se [38], e.g., not only anti-arrhythmics, but also a number of antibiotics, antifungal agents, antihistamines, and antipsychotics via different mechanisms. Moreover, most inhalational anesthetics and some neuromuscular blockers affect ventricular repolarization, often reflected by QTc interval prolongation [39], and some of our patients also underwent emergency surgeries under general anesthesia. Therefore, the potentially confounding effects of these other medications on changes in QTc interval in TBI patients requires further study.

The difference in gender was also important limitation in our study. Women commonly have longer QTc interval than men, because estrogen induces QTc prolongation whereas testosterone affects cardiac potassium channels shortening QTc [38]. In the present study, men constituted the majority of the studied population. Therefore, we can assume that sex did not significantly affect QTc interval, however this speculation requires further dedicated confirmation.

## 5. Conclusions

The present study documented the untoward effect of plasma hyperosmolality on QTc interval and cardiac rhythm in patients treated for isolated TBI. Specifically, an increase in plasma osmolality >313 mOsm/kg H_2_O significantly increased the risk for QTc prolongation >500 ms while also increasing the incidence of atrial fibrillation. Based on our findings we can speculate that mannitol does not notably affect cardiac electrical function and that electrocardiographic disorders more likely result from plasma hyperosmolality instead. We also suggest that when hyperosmolar therapy is used, that plasma osmolality not exceed 310 mOsm/kg H_2_O (rather than the currently recommended 320 mOsm/kgH2O), although this suggested new cutoff should also be corroborated via additional studies.

## Figures and Tables

**Table 1:** Demographic data of studied population. GCS—Glasgow Coma Score, SAH—subarachnoid hemorrhage, ICU—Intensive Care Unit.

Patients	Male	*n* = 72		
	Female	*n* = 22		
	GCS	4.56 ± 1.52 (min 3, max 8)		
28-day outcome	Mortality	23 (24.5%)		
	Discharged from ICU	56 (59.6%)		
	Treated longer than 28 days	15 (15.9%)		
Type of cerebral injury	Subdural/epidural hematoma	35 (37.2%)		
	Intra-cerebral haemorrhage with cerebral edema	27 (28.7%)		
	Traumatic SAH with cerebral edema	18 (19.1%)		
	Isolated cerebral edema	14 (15%)		
Mortality rate in accordance to type of injury	Subdural/epidural hematoma	2 (5.7%)		
	Intra-cerebral hemorrhage with cerebral edema	11 (40.7%)		
	Traumatic SAH with cerebral edema	7 (39.9%)		
	Isolated cerebral edema	3 (21.4%)		
Number of patients with 28-day mortality based on peak plasma osmolality range:	Below 280 mOsm/kg H_2_O	280–295 mOsm/kg H_2_O	296–310 mOsm/kg H_2_O	Above 310 mOsm/kg H_2_O
	0	0	6	17 *

* 17 non-survivors had plasma osmolality higher than 310 mOsm/kg H_2_O at least once during the study, including 3 patients with baseline plasma osmolality higher than 310 mOsm/kg H_2_O due to blood alcohol (all in group Alc), 8 patients with baseline plasma osmolality between 280–295 mOsm/kg H_2_O and 6 patients with baseline plasma osmolality below 280 mOsm/kg H_2_O, all of whom had peaks in plasma osmolality within 48 h of beginning the continuous infusion of mannitol.

**Table 2:** Factors influencing QTc interval estimated by multivariate stepwise regression analysis.

Dependent Variable	Independent	B	St. Error	β	*p*
QTc	Plasma osmolality	1.55	0.05	0.35	0.0001
	Dose of norepinephrine	32.81	0.05	0.15	0.01
	Model (R = 0.4, R^2^ = 0.19)				
